# Personality Inventory for DSM-5 in China: Evaluation of DSM-5 and ICD-11 Trait Structure and Continuity With Personality Disorder Types

**DOI:** 10.3389/fpsyt.2021.635214

**Published:** 2021-03-26

**Authors:** Shulin Fang, Zirong Ouyang, Panwen Zhang, Jiayue He, Lejia Fan, Xingwei Luo, Jianghua Zhang, Yan Xiong, Fusheng Luo, Xiaosheng Wang, Shuqiao Yao, Xiang Wang

**Affiliations:** ^1^Medical Psychological Center, The Second Xiangya Hospital, Central South University, Changsha, China; ^2^Hunan Biological and Electromechanical Polytechnic, Changsha, China; ^3^Student Affairs Department, Central South University, Changsha, China; ^4^Student Affairs Department, Central South University of Forestry and Technology, Changsha, China; ^5^Department of Human Anatomy and Neurobiology, Xiangya School of Medicine, Central South University, Changsha, China; ^6^China National Clinical Research Center on Mental Disorders (Xiangya), Changsha, China

**Keywords:** DSM-5, personality disorders, factor structure, ICD-11, dimensional trait model

## Abstract

The Personality Inventory for the DSM-5 (PID-5) is an established tool for assessing personality disorder (PD) traits that was developed based on section III of the DSM-5. It is composed of 220 items, organized into 25 facets, which are distributed among five domains. The psychometric properties of the Chinese version of the PID-5 remain to be demonstrated. Two samples were embodied in this study that included 3,550 undergraduates and 406 clinical patients. To probe the structure of the PID-5, parallel analyses were conducted to explore the unidimensionality of its 25 facets and a series of confirmatory factor analyses (CFAs) were carried out to confirm the 25 lower-order facets and their distribution among five higher-order domains. Then, the PID-5 was employed to measure the DSM-5 and ICD-11 trait models and to explore the relationship of DSM-IV categorical PDs with DSM-5 and ICD-11 personality traits. Correlation and regression analyses were conducted to probe how well DSM-IV categorical PDs correspond with maladaptive personality traits specified in the DSM-5 and five ICD-11 domains. The respective average internal reliability coefficients of the 25 facets obtained for undergraduate and clinical patient samples were 0.76 and 0.81, those obtained for the five DSM-5 domains were 0.89 and 0.91, and those obtained for the five ICD-11 domains were 0.87 and 0.89. Serial CFAs confirmed the rationality of the PID-5's lower-order 25-facet structure and higher-order five-domain structure in both samples. Correlation and regression analyses showed that DSM-5 specified traits explain the variance in PD presentation with a manifold stronger correlation (*R*^2^ = 0.24–0.44) than non-specified traits (*R*^2^ = 0.04–0.12). Overall, the PID-5 was shown to be a reliable, stable, and structurally valid assessment tool that captures pathological personality traits related to DSM-5 and ICD-11 PDs.

## Introduction

To address shortcomings in the DSM-IV categorical personality disorder (PD) diagnosis system—including complex comorbidity, within-category heterogeneity, and arbitrary diagnostic thresholds (1)—section III of the DSM-5 proposed a new hybrid alternative model of personality disorders (AMPD) that considers both pathological personality symptoms and maladaptive traits and provides accompanying assessment tools. There are two sets of core diagnostic criteria in the AMPD, A and B. Criteria A are intended to detect the severity of impairments affecting the self-domain (identity and self-direction) and the interpersonal domain (empathy and intimacy). Meanwhile, criteria B are intended to enable a dimensional assessment of pathological personality traits (that correlate with DSM-IV criteria) in concordance with the Personality Inventory for the DSM-5 (PID-5) instrument (2).

The PID-5 is a 220-item assessment organized hierarchically into 25 specific facets that are distributed among five broad domains (3). The general scoring method for the PID-5 involves the extraction of 15 facets, which are distributed three per domain across five domains (4). Although a scoring algorithm was proposed with the initial introduction of the PID-5, it was deemed to be not more suitable than the aforementioned scoring method and it has not been applied substantially in research (5). The ICD-11 proposes a fully dimensional PD diagnostic system rather than a categorical PD diagnostic system (6). This system proposes five domains—namely, negative affect, detachment, dissociality, disinhibition, and anankastia—to describe prominent PD characteristics (7), which are similar to the five domains in section III of DSM-5, with the exception of anankastia. To harmonize these two systems, scholars developed a new algorithm that uses 18 PID-5 facets to represent the five ICD-11 personality domains (8, 9).

The PID-5 has been translated into several languages since it was first published in English in 2012, starting with Italian, which Fossati et al. showed to have good reliability and validity in Italian general-population adults (10). Subsequently, the psychometric properties of German (11), French (12), Portuguese (13), Arabic (14), and Iranian (15) versions of the PID-5 were validated in normal populations. Meanwhile, German (11), Spanish (16), Dutch (17), and Danish (18) versions of the PID-5 were examined in clinical patient samples. The PID-5 has been less studied in clinical patients than in normal populations. Notwithstanding, studies have demonstrated that the PID-5 can distinguish clinical patients from normal populations effectively and that it exhibits good reliability and validity. In general, the PID-5 has been shown to be a stable and valid assessment tool across multiple language versions and various samples. Notably, almost all of these versions were validated for western populations, with the exceptions of the Arabic (14) and Iranian (15) versions. In a systematic review, McGilloway et al. (19) emphasized the importance of cultural factors in mental disorder diagnoses, particularly given that PDs are defined as enduring patterns of inner experiences and behaviors that deviate markedly from expectations, which are themselves heavily informed by cultural factors (2). To the best of our knowledge, there has been no published validation of the psychometric properties of a Chinese version of the PID-5.

The hierarchical structure of the PID-5, with its 25 lower-order facets and five higher-order domains, has been of keen interest to researchers. This construction was intended to reveal a preliminary maladaptive personality trait model for the DSM-5 and thus allow PDs to be viewed from a trait perspective (3). Based on their review of existing models and measures of maladaptive personality traits, the DSM-5 Personality and PDs workgroup identified five broad domains with which to construct a framework of the pathological personality trait model of the PID-5: negative affectivity, disinhibition, detachment, antagonism, and psychoticism (20). After considering 37 potential facets of maladaptive core personality trait features, the workgroup decided by consensus on 25 facets to be organized under the five domains (3). Several studies employing various methods, including exploratory factor analysis (EFA) (21), have confirmed the five-factor, 25-facet structure of the PID-5 (16, 17). Recently, this structure has been supported by studies of vulnerable populations, including adolescent and young-adult psychiatric patients (22). However, consistent findings of cross-loading between non-contributing facets (e.g., restricted affect, depressivity, hostility, rigid perfectionism, perseveration, and callousness) have raised questions about distinctions between contributing and non-contributing facets (16, 23). Using confirmatory factor analysis (CFA) under the condition of permitting variant correlations, Gore and Widiger confirmed a five-factor higher-order structure of the PID-5 from its 25 putative facets in a community sample (24). In general, prior EFA and CFA studies have demonstrated the appropriateness of a five-factor structure of the PID-5 directly from the 25 PID-5 facets, but the structure of the PID-5 has not been confirmed systemically starting from the level of its 220 items to first confirm the 25 specific facets before confirming the five broader domains. Additionally, the differences between contributing and non-contributing facets have not been clarified.

Section III of the DSM-5 retains six PDs from the DSM-IV, including borderline PD, schizotypal PD, antisocial PD, narcissistic PD, avoidant PD, and obsessive–compulsive PD (2) and includes descriptions corresponding to specific diagnostic traits. Corresponding traits were also specified for four PDs that were not included in section III of DSM-5 (paranoid PD, schizoid PD, histrionic PD, and dependent PD) in the “cross-walk” of the DSM-5 task force before the publication of the DSM-5 manual (25). Information from the psychiatric categorial PD diagnostic system in the DSM-IV was included in section II of the DSM-5.

To support clinical diagnostic practices, it is important to clarify how the full-dimensional PD diagnostic system relates to each PD. Although most of the proposed traits correlate strongly with corresponding PDs, some non-proposed traits show moderate or strong correlations with PDs (26–28). Indeed, a meta-analysis that was generally supportive of the specified PD traits showed that the AMPD model had low discriminative validity with some non-proposed traits correlating moderately or strongly with PDs (29). Thus, although traits that have been supposed to be related to particular PDs in the DSM-5 show a reasonable correspondence to those PDs, a further demonstration of the concrete significance of proposed traits is needed to optimize PD diagnostic methods.

It is imperative to establish how well the five domains of the full dimensional PD diagnostic system introduced in the ICD-11 capture PD categories. Previously, the five PID-5 domains have been associated with specific PD diagnoses as follows: (1) negative affect with paranoid PD, borderline PD, histrionic PD, avoidant PD, dependent PD, and obsessive–compulsive PD; (2) detachment with schizoid PD, schizotypal PD, and avoidant PD; (3) dissociality with paranoid PD, antisocial PD, borderline PD, histrionic PD, and narcissistic PD; (4) disinhibition with antisocial PD, borderline PD, and histrionic PD; and (5) anankastia with obsessive–compulsive PD (30, 31). The five DSM-5 domains are similar to those of the ICD-11 and showed similar correlation patterns, except that the psychoticism domain related mostly to paranoid PD and schizotypal PD (25).

Using the PID-5 to compare how categorical PDs relate to DSM-5 and ICD-11 domains can provide information that will be useful for harmonizing these two systems. Data regarding the relationships between these two systems are particularly lacking in relation to Chinese samples. Thus, the present study had three aims. Firstly, we examined the psychometric properties of the Chinese version of the PID-5, including its reliability and factor structure. Secondly, we used the PID-5 to investigate how the DSM-5 and ICD-11 trait models relate to each other and the PID-5. Lastly, we explored how well the DSM-5 and ICD-11 trait models fit categorical PDs determined based on the Personality Diagnostic Questionnaire (PDQ)-4+.

## Methods

### Sample

Healthy undergraduate and clinical patient samples were recruited separately. Written informed consent forms were completed by all participants. This study was approved by the ethics committee of Second Xiangya Hospital, Central South University. The demographic characteristics of the subjects in each group and the diagnoses of the patients are shown in Table 1.

**Table 1:** Sample characteristics by group.

**Characteristic**	**Undergraduate sample *N* = 3,550**	**Clinical sample *N* = 406**
Male/female gender ratio, *N* (%)	1,477 (41.6%)/2,324 (58.7%)	155 (38.2%)/251 (61.8%)
Mean age ± SD, years	18.25 ± 0.93	24.48 ± 8.25
Age range, years	18–23	18–57
**Diagnosis**, ***N*** **(%)**		
Depressive disorder	–	157 (38.67%)
Bipolar disorder	–	30 (7.39%)
Schizophrenia	–	21 (5.17%)
Obsessive–compulsive disorder	–	27 (6.65%)
Anxiety disorder	–	38 (9.36%)
Personality disorder	–	125 (30.79%)
Others	–	8 (1.97%)

For the undergraduate sample, we conducted random recruitment of 3,800 freshmen from two universities in Hunan Province to complete the PID-5 and PDQ-4+. After exclusion of questionnaires that were unfinished or that had garbled responses, valid completed questionnaires were received from 3,550 of the recruits, including 1,447 males (41.6%) and 2,073 females (58.4%) with a mean age of 18.25 ± 0.93 years. A random subsample of 250 students was retested 4 weeks later; valid retest data were obtained from 204 of them, including 82 males (40.2%) and 122 females (59.8%), after deletion of invalid and extreme data.

The clinical sample included 406 patients recruited from the psychiatric outpatient clinic of the Second Xiangya Hospital. The patient sample included 155 males (38.2%) and 251 females (61.8%) with a mean age of 24.48 ± 8.25 years. All patients were diagnosed by two psychiatrists according to the DSM-IV. Their diagnoses included major depressive disorder, bipolar disorder, schizophrenia, obsessive–compulsive disorder, anxiety disorder, and PDs (see Table 1).

### Instruments

#### PID-5

The PID-5 is a 220-item scale intended for individuals who are at least 18 years old. The items constitute 25 facets, which are organized into five domains (negative affectivity, detachment, antagonism, disinhibition, and psychoticism). Each item is answered on a four-point Likert-type scale ranging from 0 (very false or often false) to 3 (very true or often true); some are reverse coded. Each domain subscore is calculated as the average of its three contributing facets following the guidance provided by the DSM-5 (4). The remaining facets not included in domain subscore calculations are called non-contributing facets. The Chinese version of the PID-5 used in our study was developed through the translation/back-translation method wherein two linguistic experts translated the English version into a Chinese version, which was then back-translated by another translator who was unaware of the original PID-5. The back-translated version was compared with the original version to complete the Chinese version.

#### PDQ-4+

The original PDQ-4+ is a 99-item true–false instrument; the contents of the items correspond directly with the DSM-IV criteria for PDs (32). In the DSM-IV, there are 10 PD types in three clusters. Cluster A includes paranoid PD, schizoid PD, and schizotypal PD. Cluster B includes borderline PD, antisocial PD, narcissistic PD, and histrionic PD. Cluster C includes avoidant PD, obsessive–compulsive PD, and dependent PD. We employed Ling, Qian, and Yang's 108-item Chinese version of the PID-5, which has been adapted to Chinese culture (consistency reliability coefficient = 0.70–0.87; retest reliability coefficient = 0.50–0.80) (33).

### Analysis

#### Reliability

Internal reliability and retest reliability statistics were completed in SPSS 25.0 software (34). Internal reliability was represented by Cronbach's α coefficient and mean inter-item correlation (MIC) values. We considered Cronbach's α coefficients above 0.70 to be acceptable and above 0.60 to be borderline acceptable (35). The optimal MIC range was 0.10–0.40 (36). Facet and domain score stability over the test–retest interval was assessed with Spearman correlation analysis (37).

#### Construct Validity

To probe facet structure, parallel analyses were conducted in M-plus 7.0 (38), which enabled us to determine how many factors to extract for each facet. Eigenvalues obtained from a factor analysis of the actual data were compared to eigenvalues obtained from a factor analysis of a random dataset (3,500 random permutations of the original dataset), and the number of factors to be retained was determined based on the number of actual-dataset eigenvalues that exceeded the upper 95% confidence limit of the random-dataset eigenvalues (39).

Next, we conducted a series of CFAs with a maximum likelihood with robust standards errors (MLR) for the 25 lower-order facets and the five higher-order domains of the PID-5 (40). We chose the contributing facets of each domain when confirming the five-factor structure of the PID-5 for two reasons. First, we used the domain-contributing facets when we calculated domain scores according to DSM-5 guidelines. Secondly, the non-contributing facets demonstrated cross-loading on domains yielding a blended structure (41).

The CFAs were conducted in M-plus 7.0 (38). Model fit was evaluated based on comparative fit index (CFI), standardized root mean square residual (SRMR), and root mean square error of approximation (RMSEA) values with the following criteria for a good fit: CFI ≥0.90, RMSEA ≤0.08, and SRMR ≤0.08 (42). Owing to the complexity and stability of the PID-5, parceling was applied to simplify and confirm the factor structure (43). A total of 61 four-item sets were examined.

**Figure 1:**
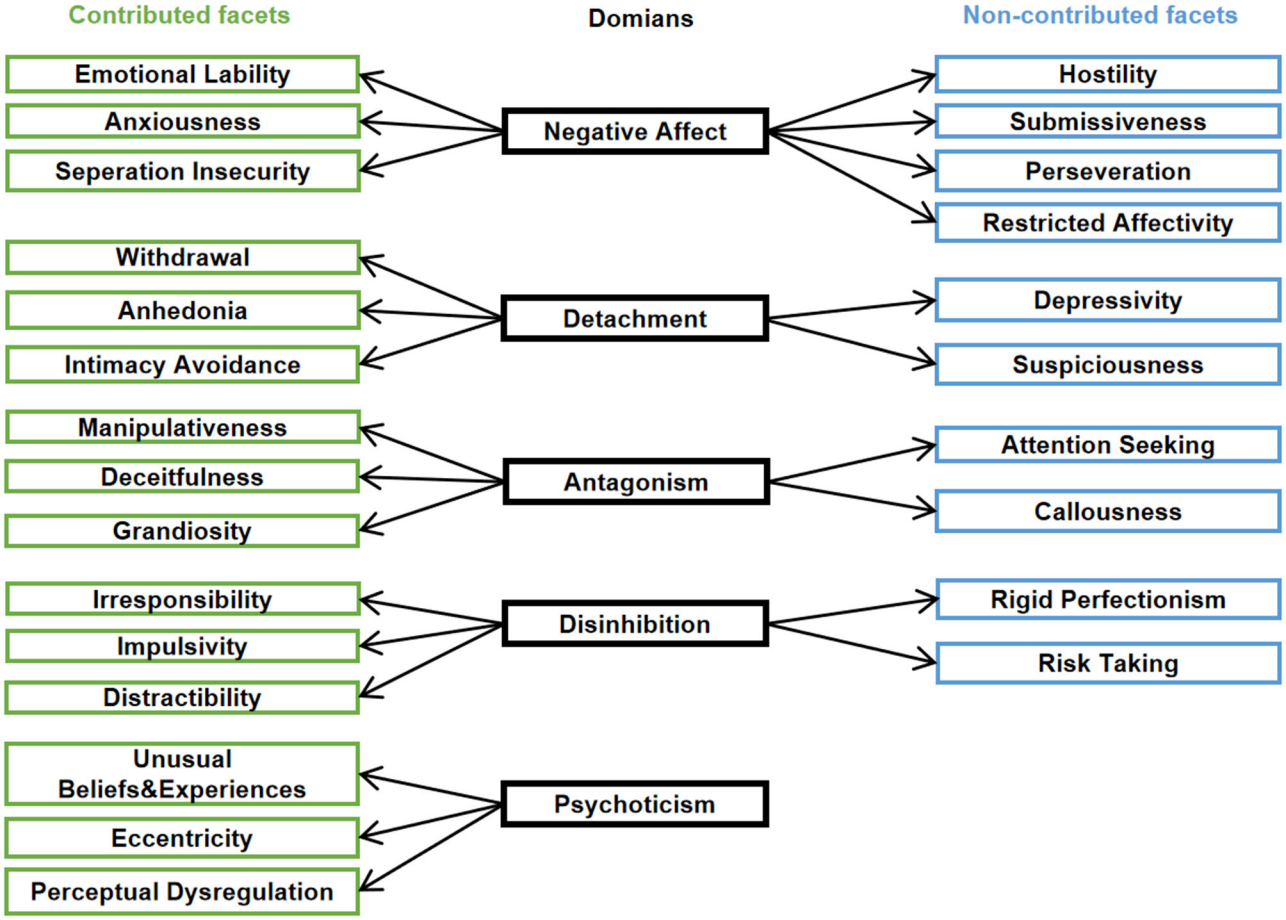
The PID-5 structure. The domains are shown in the middle column, with contributed facets on the left (green) and non-contributed facets (blue) on the right, respectively.

The initial structure proposed in which each domain contains three to seven facets (3) and the concrete structure of the PID-5 used in our study are shown in Figure 1. Each domain subscore was determined by three contributing facets indicated by the DSM-5 (4). The structure of the first and second columns in Figure 1 was subjected to CFA of the higher-order structure of the PID-5.

#### Relationships Among PDQ-4+-Based PDs, Section III of the DSM-5, and the ICD-11

To probe the relationship between section III of the DSM-5 and the ICD-11, Spearman correlation analyses were conducted between the 25 facets and five domains in section III of the DSM-5 and the five ICD-11 domains. Spearman correlation analyses were also conducted between the 10 PDQ-4+-based PDs and the 25 facets and five domains of the PID-5, as well as between the 10 PDs and the five domains of the ICD-11. Correlation coefficients >0.30 were considered acceptable (44).

Regression analyses were carried out in two blocks to determine the capacity of pathological PD traits from section III of the DSM-5 to predict PDQ-4+ score variance. Proposed traits were entered in block 1 and non-proposed traits were entered in block 2. Additionally, the five DSM-5 domains and the five ICD-11 domains were entered into a regression analysis to determine the degree to which personality traits from the ICD-11 and DSM-5 explain the variance of the 10 PDs.

## Results

### Reliability

Descriptive analysis affirmed normal distributions of the 25 facets and five domains of the PID-5, in both the undergraduate sample and the clinical patient sample (means are reported with standard deviations in (Supplementary Tables 1, 2). Independent *t*-tests showed that the PID-5 facets and domains were distributed differently between the undergraduate sample and the PDs sample (Supplementary Table 3).

Regarding facet reliability of the 25 PID-5 facets (Supplementary Table 4), with the exceptions of suspiciousness and restricted affectivity, the Cronbach's α coefficients of the remaining 23 facets in the undergraduate sample were all >0.65, with a mean coefficient value of 0.76 (range, 0.54–0.91). With the exception of restricted affectivity, the Cronbach's α coefficients of the remaining 24 facets in the clinical patient sample were >0.70, with a mean coefficient value of 0.81 (range, 0.53–0.93). The mean MIC values for the 25 facets were 0.29 (range, 0.14–0.43) and 0.24 (range, 0.22–0.27) in the undergraduate sample and clinical patient sample, respectively. The mean retest reliability coefficient of the facets in the undergraduate sample was 0.68 (range, 0.52–0.81).

For the five domains from section III of the DSM-5, the mean Cronbach's α coefficient values were 0.89 (range, 0.86–0.93) in the undergraduate sample and 0.91 (range, 0.87–0.95) in the clinical patient sample (Table 2). The associated mean MIC values were 0.24 (0.22–0.27) in the undergraduate sample and 0.30 (range, 0.24–0.34) in the clinical patient sample. The mean retest reliability coefficient of the facets in the clinical patient sample was 0.81 (range, 0.78–0.86).

**Table 2:** Internal reliability and retest reliability of DSM-5 and ICD-11 domains.

	**Internal reliability**				
**Domain**	**Undergraduate sample**		**Clinical patient sample**		**Retest reliability**
	**α**	**MIC**	**α**	**MIC**	
**DSM-5**					
Negative affect	0.88	0.24	0.91	0.30	0.81^**^
Detachment	0.88	0.24	0.92	0.33	0.78^**^
Antagonism	0.86	0.22	0.87	0.24	0.78^**^
Disinhibition	0.88	0.25	0.90	0.30	0.81^**^
Psychoticism	0.93	0.27	0.95	0.34	0.86^**^
**ICD-11**					
Negative affect	0.93	0.21	0.95	0.28	0.83^**^
Detachment	0.85	0.21	0.89	0.26	0.86^**^
Dissociality	0.89	0.20	0.90	0.21	0.83^**^
Disinhibition	0.84	0.13	0.87	0.16	0.82^**^
Anankastia	0.84	0.22	0.86	0.25	0.78^**^

*The α-values are Cronbach's α coefficients. MIC, mean inter-item correlations*.

** *p < 0.01*.

For the five domains from the ICD-11, we obtained mean Cronbach's α coefficient values of 0.87 (range, 0.84–0.93) in the undergraduate sample and 0.89 (range, 0.86–0.95) in the clinical patient sample (Table 2). The associated mean MIC values were 0.19 (0.13–0.22) and 0.23 (range, 0.16–0.28), respectively. The mean retest reliability coefficient of the facets in the clinical patient sample was 0.82 (range, 0.78–0.86).

### Construct Validity

#### Unidimensionality

Parallel comparative analyses were conducted to determine the unidimensionality of the 25 facets of the PID-5 in the undergraduate sample and clinical patient sample, in which empirical data eigenvalues exceeding the upper 95% confidence limit of random data eigenvalues were considered reasonable to retain as factors. For the undergraduate sample, parallel analyses identified 17 unidimensional and 7 bi-dimensional facets (depressivity, hostility, perseveration, restricted affectivity, rigid perfectionism, suspiciousness, and withdrawal), and risk-taking suggested retaining three factors.

For the clinical patient sample, three facets (hostility, perseveration, and restricted affectivity) were suggested to retain two factors and one facet (restricted affective) was suggested to retain three factors. Overall, those facets identified as non-unidimensional tended to relate to non-contributing facets in both the undergraduate sample and the clinical patient sample.

#### Factor Structure

Serial CFAs showed that both the lower-order 25-facet model and the higher-order five-domain model fit well in the undergraduate and clinical patient samples. The CFI, SRMR, and RMSEA values obtained in the CFAs are reported in Table 3. Briefly, with the undergraduate sample, the fit indices for the lower-order model and higher-order five-domain model met the criteria for a reasonable fit. With the clinical patient sample, the CFA results for the lower-order structure showed a good fit, while those for the higher-order five-domain model met the criteria for a reasonable fit.

**Table 3:** Confirmatory factor analysis results.

**Sample**	**χ^2^**	***df***	**CFI**	**SRMR**	**RMSEA (90%CI)**
**Undergraduates**					
Lower order	8134.037	1,469	0.935	0.046	0.036 (0.035, 0.037)
Higher order	5691.261	535	0.907	0.058	0.052 (0.051, 0.053)
**Clinical patients**					
Lower order	2758.241	1,469	0.919	0.049	0.046 (0.044, 0.049)
Higher order	1380.865	535	0.900	0.078	0.062 (0.058, 0.066)

*χ^2^, Chi-square; df , degrees of freedom; CFI, comparative fit index; SRMR, standardized root mean square residual; RMSEA, root mean square error of approximation*.

### Relationship Between Domains of the DSM-5/Section III and Domains of the ICD-11

Four domains correlated strongly between the two systems in both the undergraduate sample and the clinical patient sample (DSM-5, section III: negative affect, detachment, antagonism, and disinhibition; ICD-11: negative affect detachment, dissociality, and disinhibition). The correlation coefficients are reported in Table 4. Briefly, the coefficients for similarity of related domains ranged from 0.89 to 0.94 in the undergraduate sample and ranged from 0.89 to 0.95 in the clinical patient sample. Meanwhile, the coefficients for other correlations ranged from 0.27 to 0.69 in the undergraduate sample and ranged from 0.30 to 0.71 in the clinical patient sample.

**Table 4:** Correlations between DSM-5 section III domains and ICD-11 domains.

**DSM-5 domain**	**ICD-11 domain, undergraduate sample/clinical patient sample**				
	**Negative affect**	**Detachment**	**Dissociality**	**Disinhibition**	**Anankastia**
Negative affect	0.89^**^/0.89^**^	0.32^**^/0.38^**^	0.46^**^/0.36^**^	0.56^**^/0.60^**^	0.62^**^/0.56^**^
Detachment	0.61^**^/0.67^**^	0.91^**^/0.92^**^	0.27^**^/0.12^**^	0.42^**^/0.47^**^	0.45^**^/0.40^**^
Antagonism	0.53^**^/0.40^**^	0.30^**^/0.23^**^	0.89^**^/0.89^**^	0.45^**^/0.36^**^	0.45^**^/0.48^**^
Disinhibition	0.67^**^/0.71^**^	0.40^**^/0.46^**^	0.41^**^/0.30^**^	0.94^**^/0.95^**^	0.39^**^/0.36^**^
Psychoticism	0.69^**^/0.64^**^	0.51^**^/0.54^**^	0.58^**^/0.46^**^	0.56^**^/0.56^**^	0.61^**^/0.58^**^

*Correlations between similar domains from the two systems are in bold*;

** *p < 0.01*.

### Relationships of PDQ-4+-Based PDs With Section III of the DSM-5 and the ICD-11

#### Section III of the DSM-5

As reported in Table 5, correlation analysis showed that, in general, specified traits correlated more strongly with PDQ-4+ scores for their respective PDs (mean *r*^2^ = 0.38) than did non-specified traits (mean *r*^2^ = 0.19). However, some specified traits correlated weakly with putatively corresponding PDs. For example, the correlation coefficient between avoidant PD and intimacy avoidance was 0.15, which is lower than the highest value coefficient obtained for non-specified traits (i.e., coefficient for borderline PD with anhedonia was 0.40).

**Table 5:** Correlation between pathological personality traits measured by the PID-5 and personality disorders measured by PDQ-4+ in the undergraduate sample.

**Trait**	**PPD**	**SPD**	**STPD**	**BPD**	**ASPD**	**NPD**	**HPD**	**APD**	**OCPD**	**DPD**
**25 facets**										
Anhedonia	0.26^*^	0.35^*^	0.20^*^	0.40^*^	0.10^*^	0.22^*^	0.04^*^	0.43^*^	0.21^*^	0.30^*^
Anxiousness	0.42^*^	0.19^*^	0.34^*^	0.50^*^	0.15^*^	0.38^*^	0.28^*^	0.50^*^	0.40^*^	0.46^*^
Attention seeking	0.33^*^	−0.04^*^	0.22^*^	0.23^*^	0.24^*^	0.42^*^	0.50^*^	0.21^*^	0.24^*^	0.27^*^
Callousness	0.32^*^	0.25^*^	0.25^*^	0.33^*^	0.31^*^	0.30^*^	0.18^*^	0.24^*^	0.16^*^	0.20^*^
Cognitive and perceptual dysregulation	0.38^*^	0.25^*^	0.41^*^	0.52^*^	0.28^*^	0.40^*^	0.27^*^	0.38^*^	0.35^*^	0.38^*^
Deceitfulness	0.38^*^	0.10^*^	0.26^*^	0.32^*^	0.34^*^	0.35^*^	0.26^*^	0.26^*^	0.14^*^	0.27^*^
Depressivity	0.33^*^	0.30^*^	0.30^*^	0.51^*^	0.20^*^	0.29^*^	0.15^*^	0.47^*^	0.30^*^	0.38^*^
Distractibility	0.23^*^	0.15^*^	0.16^*^	0.41^*^	0.20^*^	0.23^*^	0.24^*^	0.40^*^	0.21^*^	0.41^*^
Eccentricity	0.29^*^	0.28^*^	0.43^*^	0.40^*^	0.31^*^	0.35^*^	0.20^*^	0.27^*^	0.31^*^	0.21^*^
Emotional lability	0.34^*^	0.22^*^	0.26^*^	0.57^*^	0.26^*^	0.38^*^	0.39^*^	0.39^*^	0.31^*^	0.38^*^
Grandiosity	0.32^*^	0.09^*^	0.31^*^	0.20^*^	0.23^*^	0.43^*^	0.32^*^	0.16^*^	0.24^*^	0.16^*^
Hostility	0.47^*^	0.22^*^	0.34^*^	0.47^*^	0.30^*^	0.44^*^	0.34^*^	0.39^*^	0.31^*^	0.33^*^
Impulsivity	0.19^*^	0.10^*^	0.09^*^	0.43^*^	0.33^*^	0.24^*^	0.29^*^	0.27^*^	0.14^*^	0.30^*^
Intimacy avoidance	0.07^*^	0.38^*^	0.13^*^	0.15^*^	−0.02	0.06^*^	−0.04^*^	0.15^*^	0.20^*^	0.07^*^
Irresponsibility	0.23^*^	0.15^*^	0.15^*^	0.38^*^	0.28^*^	0.24^*^	0.20^*^	0.32^*^	0.12^*^	0.34^*^
Manipulativeness	0.35^*^	0.06^*^	0.29^*^	0.21^*^	0.27^*^	0.34^*^	0.32^*^	0.12^*^	0.18^*^	0.16^*^
Perseveration	0.33^*^	0.24^*^	0.33^*^	0.44^*^	0.20^*^	0.36^*^	0.27^*^	0.40^*^	0.41^*^	0.41^*^
Restricted affect	0.21^*^	0.33^*^	0.28^*^	0.21^*^	0.11^*^	0.20^*^	−0.02	0.28^*^	0.24^*^	0.16^*^
Rigid perfectionism	0.31^*^	0.18^*^	0.35^*^	0.24^*^	0.04	0.33^*^	0.22^*^	0.26^*^	0.50^*^	0.24^*^
Risk taking	0.03	−0.05^*^	0.06^*^	0.06^*^	0.39^*^	0.11^*^	0.12^*^	−0.13^*^	−0.03	−0.12^*^
Separation insecurity	0.29^*^	−0.02	0.18^*^	0.32^*^	0.12^*^	0.28^*^	0.27^*^	0.36^*^	0.21^*^	0.45^*^
Submissiveness	0.19^*^	0.07^*^	0.16^*^	0.29^*^	0.04	0.18^*^	0.22^*^	0.37^*^	0.22^*^	0.42^*^
Suspiciousness	0.44^*^	0.21^*^	0.33^*^	0.35^*^	0.19^*^	0.34^*^	0.19^*^	0.34^*^	0.27^*^	0.27^*^
Unusual belief and experiences	0.35^*^	0.16^*^	0.47^*^	0.33^*^	0.25^*^	0.37^*^	0.23^*^	0.19^*^	0.25^*^	0.17^*^
Withdrawal	0.25^*^	0.45^*^	0.33^*^	0.35^*^	0.10^*^	0.25^*^	0.01	0.41^*^	0.30^*^	0.22^*^
**DSM-5 domains**										
Negative affect	0.44^*^	0.17^*^	0.34^*^	0.58^*^	0.22^*^	0.43^*^	0.39^*^	0.52^*^	0.39^*^	0.54^*^
Detachment	0.25^*^	0.50^*^	0.30^*^	0.39^*^	0.08^*^	0.23^*^	0.01	0.43^*^	0.30^*^	0.26^*^
Antagonism	0.42^*^	0.10^*^	0.34^*^	0.30^*^	0.35^*^	0.44^*^	0.35^*^	0.23^*^	0.21^*^	0.25^*^
Disinhibition	0.26^*^	0.16^*^	0.16^*^	0.49^*^	0.31^*^	0.28^*^	0.29^*^	0.41^*^	0.19^*^	0.43^*^
Psychoticism	0.39^*^	0.28^*^	0.50^*^	0.49^*^	0.33^*^	0.43^*^	0.27^*^	0.34^*^	0.34^*^	0.30^*^
**ICD-11 domains**										
Negative affect	0.51^*^	0.29^*^	0.40^*^	0.65^*^	0.28^*^	0.50^*^	0.36^*^	0.53^*^	0.43^*^	0.47^*^
Detachment	0.21^*^	0.49^*^	0.31^*^	0.29^*^	0.07^*^	0.21^*^	−0.02	0.35^*^	0.32^*^	0.19^*^
Dissociality	0.43^*^	0.09^*^	0.34^*^	0.34^*^	0.33^*^	0.50^*^	0.45^*^	0.23^*^	0.28^*^	0.26^*^
Disinhibition	0.21^*^	0.13^*^	0.16^*^	0.47^*^	0.42^*^	0.32^*^	0.31^*^	0.32^*^	−0.19^*^	0.35^*^
Anankastia	0.37^*^	0.24^*^	0.39^*^	0.41^*^	0.14^*^	0.41^*^	0.29^*^	0.38^*^	0.53^*^	0.37^*^

*In the first section (facets), coefficients between specified traits and corresponding PDs are in bold. In the DSM-5 and ICD-11 domain sections, hypothesized correlations are in bold*.

* *p < 0.01. (P/S/ST/B/AS/N/H/A/OC/D) PD refer to (paranoid/schizoid/schizotypal/borderline/antisocial/narcissistic/histrionic/avoidant/obsessive-compulsive/dependent) personality disorder, respectively*.

As reported in Table 6, regression analysis showed that PD-specified traits (block 1) were predictive of the variance of their corresponding PDs, with coefficients ranging from 0.24 to 0.41 (for explicit beta coefficients, see Supplementary Table 5). However, the traits manipulativeness and irresponsibility were not predictive of antisocial PD criteria, and the traits separation insecurity and risk-taking were not predictive of borderline PD criteria. Non-specified traits (block 2) provided only minor incremental information for all 10 PDs, with explained variance values ranging from 0.02 to 0.12. Specified and non-specified traits were related to PD criteria scores with explained variance values ranging from 0.29 to 0.46.

**Table 6:** Regression analysis results for PID-5 specified and non-specified traits' predictiveness of SCID-II PD criteria scores in undergraduate sample.

**Personality disorder**	**Overall *R*^**2**^**	**Δ*****R***^****2****^	
		**Criterion B traits**	**Non-specified traits**
Paranoid	0.35^**^	0.30^**^	0.05^**^
Schizoid	0.29^**^	0.26^**^	0.03^**^
Schizotypal	0.33^**^	0.28^**^	0.05^**^
Antisocial	0.29^**^	0.27^**^	0.02^**^
Borderline	0.46^**^	0.44^**^	0.02^**^
Histrionic	0.35^**^	0.31^**^	0.04^**^
Narcissistic	0.36^**^	0.24^**^	0.12^**^
Avoidant	0.38^**^	0.33^**^	0.05^**^
Dependent	0.37^**^	0.32^**^	0.05^**^
Obsessive–compulsive	0.33^**^	0.29^**^	0.04^**^

** *p < 0.01*.

The correlation coefficients obtained for analyses between the five DSM-5 domains and the 10 PDs ranged from 0.01 to 0.58 with a mean value of 0.33 (Table 5). Regression analysis showed that all five domains together predicted variance of the 10 PDs with a mean coefficient value of 0.28 (range, 0.18–0.42) (Table 7). Each domain was found to have focused correlations with PDs. For example, disinhibition correlated more strongly with borderline PD, antisocial PD, and dependent PD than with other PDs.

**Table 7:** Multiple regression coefficients for DSM-5 and ICD-11 domains as predictors of 10 PD.

**Domain**	**PPD**	**SPD**	**STPD**	**BPD**	**ASPD**	**NPD**	**HPD**	**APD**	**OCPD**	**DPD**
**DSM-5**										
Negative affect	0.34^**^	−0.04	0.14^**^	0.42^**^	−0.06^**^	0.30^**^	0.32^**^	0.43^**^	0.30^**^	0.47^**^
Detachment	0.05^**^	0.53^**^	0.11^**^	0.07^**^	−0.18^**^	−0.02	−0.28^**^	0.26^**^	0.17^**^	0.00
Antagonism	0.28^**^	−0.08^**^	0.09^**^	**−0.01**	0.21^**^	0.26^**^	0.24^**^	−0.01	−0.01	0.02^**^
Disinhibition	−0.13^**^	−0.13^**^	−0.26^**^	0.13^**^	0.24^**^	−0.06	0.12^**^	0.06^**^	−0.16^**^	0.18^**^
Psychoticism	0.07^**^	0.14^**^	0.48^**^	0.15^**^	0.21^**^	0.16^**^	0.02	−0.08^**^	0.21^**^	−0.09^**^
*R*^2^	0.28^**^	0.27^**^	0.29^**^	0.42^**^	0.18^**^	0.30^**^	0.24^**^	0.33^**^	0.22^**^	0.31^**^
**ICD-11**										
Negative affect	0.49^**^	0.12^**^	0.26^**^	0.59^**^	0.04^**^	0.34^**^	0.22^**^	0.49^**^	0.19^**^	0.37^**^
Detachment	−0.05^**^	0.46^**^	0.12^**^	−0.05^**^	−0.09^**^	−0.07^**^	−0.28^**^	0.10^**^	0.08^**^	−0.10^**^
Dissociality	0.24^**^	−0.08^**^	0.17^**^	−0.04*	0.22^**^	0.32^**^	0.33^**^	−0.09^**^	−0.01	−0.05^**^
Disinhibition	−0.16^**^	−0.08^**^	−0.17^**^	0.12^**^	0.35^**^	−0.04^*^	0.09^**^	−0.00	−0.10^**^	0.11^**^
Anankastia	0.03	0.02	0.14^**^	0.04^*^	−0.08^**^	0.08^**^	0.07^**^	0.08^**^	0.41^**^	0.16^**^
*R*^2^	0.31^**^	0.25^**^	0.23^**^	0.43^**^	0.21^**^	0.33^**^	0.28^**^	0.30^**^	0.30^**^	0.25^**^

*Hypothesized correlations are in bold*;

* p < 0.05 and

** *p < 0.001*.

#### ICD-11

The correlation coefficients, beta coefficients, and *R*^2^ values obtained in our examination of the relationship of ICD-11 domains with PDs are reported in Tables 5, 7. The five ICD-11 domains correlated with PDs in distinct patterns. The mean correlation coefficient for hypothesized traits was 0.43 (bold values in Table 6; hypotheses based on a previous study), and the mean coefficient was 0.28 for non-hypothesized traits. With the exception of the correlation between detachment and paranoid PD (*r*^2^ = 0.21), coefficient values were greater for hypothesized correlations than for non-hypothesized traits. Unexpectedly, some non-hypothesized correlations showed relatively high correlation coefficients (e.g., negative affect-schizotypal PD coefficient = 0.40). Regression analysis showed that the five ICD-11 domains together predicted the variance of PDs with coefficients ranging from 0.21 to 0.43.

## Discussion

In the present study, we obtained comprehensive data supporting the reliability and validity of the Chinese version of the PID-5 in undergraduate and clinical patient samples. To the best of our knowledge, this study provides the first systematic demonstration of the structure of the PID-5 across three levels: each item-composed facet structure, lower-order 25-facet structure, and higher-order five-domain structure. Previous studies have included one or two levels. Furthermore, our results indicate that there was a smooth transition from the categorical paradigm of the DSM-IV to the dimensional paradigms of the DSM-5/section III and ICD-11.

Concerning reliability, our results showed internal reliability coefficients above 0.85 in both the undergraduate sample and the clinical patient sample, together with retest reliability coefficients above 0.75 for all five domains, indicating that the PID-5 is a reliable and stable tool for assessing pathological PD traits in individuals with a Chinese cultural background. Moreover, to the best of our knowledge, only three prior studies of the PID-5 included retest reliability (13, 45, 46). Thus, this study fills a gap in available evidence regarding the stable reliability of the PID-5. A previous psychometric review of the PID-5 underscored the importance of test–retest research due to measurement differences between retest reliability and internal reliability (47).

On the facet level, the internal coefficients that we obtained for the restricted affectivity facet were relatively low compared with those obtained for other facets in both samples, which may be the consequence of cultural divergence. Generally, modesty and restrained affect are more integral to and much more strongly promoted in Chinese culture than in western cultures, and most of the PID-5 literature has been conducted in the context of western cultures (48). The internal coefficient obtained for the facet suspiciousness was also relatively low in our undergraduate sample, perhaps due, at least in part, to two of the items that this facet is based on being reverse coded. Reverse coding can alter response patterns, compared with forward-coded items, and thus may impede internal reliability (21, 49).

The present results confirmed the 25-facet lower-order structure of the PID-5, as well as the five-domain higher-order structure of the PID-5 after examination of the unidimensionality of the 25 facets. Our unidimensionality analysis of PID-5 facets affirmed the distinction between contributing and non-contributing facets. Specifically, 7 of 10 non-contributing facets in the undergraduate sample and 4 of 10 non-contributing facets in the clinical patient sample were demonstrated to be non-dimensional. These findings are consistent with prior research, including a Czech study showing non-dimensionality of the non-contributed facets of callousness, risk-taking, depressivity, and suspiciousness (41), as well as a French study reporting non-dimensionality of callousness and depressivity (12). Additionally, prior exploratory factor analysis studies of the 25 PID-5 facets have shown that some facets exhibit cross-loading over the five-factor higher-order structure [e.g., rigid perfectionism loading primarily on psychoticism instead of disinhibition; (22)]. Some facets exhibit pure relationships with their corresponding domains, while others share meaningful features across domains (50, 51), which is reflected in the DSM-5 domain scoring rubric. Our parallel analysis suggested that two factors should be retained in the withdrawal facet in the undergraduate sample, one that represents an attitude of affiliation distancing and another that represents one's motivation for affiliation; notably, these two factors appear to be conceptually distinct in Chinese culture (52). Although the distinction between contributing and non-contributing facets has been affirmed, there remains a need to explore the significance of specified vs. non-specified traits in the PID-5 structure.

The presently reported series of CFAs demonstrated the established framework of the PID-5. Consistent with prior findings reported for the Italian PID-5 (10) and the Arabic PID-5 (14), we confirmed that the Chinese PID-5 has a five-domain higher-order structure in both normal and clinical samples. However, these prior studies did not demonstrate the 25-facet lower-order structure, which represents the core markers of the domains and is integral to the overall structure of the PID-5 (3). Thus, here we report, for the first time to our knowledge, a comprehensive, systemic study of the structure of the PID-5 that demonstrates the validity of the structure of the PID-5.

We observed obviously distinct correlation patterns between the DSM-5/section III domains and the ICD-11 domains, particularly for four similar domains across diagnostic systems. Overall, we found that the PID-5 represents the five domains of the ICD-11 accurately. Comparing the ICD-11 with section III of the DSM-5, the ICD-11 facet anankastia, which encompasses perfectionism and emotional and behavioral constraint, seems beneficial for capturing the main characteristics of obsessive–compulsive PD. Although psychoticism is not included in the ICD-11 because it considers the schizotypal phenotype to be a variant of schizophrenia rather than a distinct PD, a series of studies have highlighted the importance of psychoticism in PD descriptions (53, 54). Overall, harmonizing PD concepts between section III of the DSM-5 and the ICD-11 would benefit from more accurate elaboration of PDs. The algorithm for PID-5 may be helpful in advancing the harmonizing process.

Elucidating the relationships of the two presently examined dimensional PD diagnostic systems with a categorical PD diagnostic system can provide clinically useful information. Our correlation and regression analysis findings showing that DSM-5/section III–specified traits that have diagnostic significance for particular PDs can be discriminated from non-specified traits are consistent with previous studies to some extent (17, 26, 28, 55). However, some specified facets of avoidant PD and borderline PD had only weak correlations with their corresponding PDs and were not reliable predictors of corresponding scale scores, perhaps due to substantial overlap between facets. For example, the facet risk-taking was not as predictive of borderline PD as expected and there was substantial overlap between impulsivity and risk-taking. Accordingly, it may be that impulsivity levels may provide a sufficient representation of the disinhibited aspects of borderline PD (56).

Consistent with a previous study (8), we found that four domains that are similar between the DSM-5 and ICD-11 showed similarity-focused correlations with PDs. Although both DSM-5 and ICD-11 domains related to PDs, the domains lack robust inter-PD discriminative validity. The psychoticism domain of the DSM-5 and the anankastia domain of the ICD-11 correlated very strongly with schizotypal PD and obsessive–compulsive PD, respectively; as expected, some other non-hypothesized correlations of moderate strength were also observed. Non-hypothesized correlations may be consequent to some comorbidity across PDs. Clark et al. (57) suggested that adopting PD-specified traits as the only PD diagnostic criterion would be effective and clinically useful. However, full-dimensional diagnosis takes time. Hence, when defining the particular traits of a PD, it is important to capture the core characteristics of the PD, both conceptually and based on previous empirical findings obtained in the context of other trait models, and in so doing to consider descriptive clinical perspectives (58).

This study had some limitations that should be considered and addressed in future research. First, this study focused mainly on measured properties of the PID-5; future research should explore the external validity of the PID-5. Second, our examination of the PD diagnostic transition from the DSM-IV to the DSM-5 was limited to trait criteria for PDs without consideration of other criteria, such as impairments in self and interpersonal functioning. Third, our clinical sample was slightly but significantly older than our healthy sample (*p* < 0.01). We do not believe that this difference affected our findings given that the main purpose of this study was to explore the psychometric properties not to compare the PID-5 across groups. Finally, we used the PID-5 to assess ICD-11 domains, although there is a different personality inventory for ICD-11 developed to assess ICD-11 trait domains (59). It may be useful to conduct similar analyses as those reported here with the ICD-11 personality inventory.

## Conclusion

The present work extends prior research examining the psychometric properties of the PID-5 and does so for the first time in a Chinese sample. The results demonstrate that the PID-5 is a valid tool for assessing DSM-5 and ICD-11 pathological personality traits in Chinese individuals. More specifically, our systematic analysis confirmed, in series, the item-facet, lower-order 25-facet structure, and higher-order five-domain structure of the PID-5. The present findings highlight the difference between contributing facets and non-contributing facets and provide additional knowledge about the structure of the PID-5. Furthermore, employing 18 PID-5 facets, we assessed the five-dimensional traits of the ICD-11 and explored the relationship between the dimensional systems of the DSM-5/section III and the ICD-11, as well as their corresponding relationships with categorical PDs (DSM-IV), and thus demonstrated validity of the PID-5 while also obtaining evidence for improving trait-based criteria. Overall, the present study provided empirical evidence for the DSM-5 and ICD-11 trait models in a Chinese population.

## Data Availability Statement

The raw data supporting the conclusions of this article will be made available by the authors, without undue reservation.

## Ethics Statement

The studies involving human participants were reviewed and approved by Second Xiangya Hospital. The patients/participants provided their written informed consent to participate in this study.

## Author Contributions

SF and ZO conceptualized this study and accomplished primarily data analysis and writing. XianW, SY, and XiaoW supervised the research and revised the initial draft. PZ and JH provided the substantial analysis technical support. LF, XL, JZ, YX, and FL were responsibility for data curation. All authors contributed to the article and approved the submitted version.

## Conflict of Interest

The authors declare that the research was conducted in the absence of any commercial or financial relationships that could be construed as a potential conflict of interest.
