# Supplementary material for: Personality Inventory for DSM-5 in China: Evaluation of DSM-5 and ICD-11 Trait Structure and Continuity With Personality Disorder Types
Source: Front Psychiatry. 2021 Mar 26;12:635214. doi: 10.3389/fpsyt.2021.635214 (PMC8033014; doi:10.3389/fpsyt.2021.635214)
Supplement: Supplementary file 1 [file Data_Sheet_1.PDF]

Supplementary Material

Personality Inventory for DSM-5 (PID-5) in China: Evaluation of DSM-5 and ICD-11 Trait Structure and Continuity with Personality Disorder Types

Contents

Table 1 Descriptive analysis for 25 facets and 5 domains of the PID-5 in undergraduate sample

Table 2 Descriptive analysis for 25 facets and 5 domains of the PID-5 in clinical patient sample

Table3 The t-test between undergraduate sample and personality disorders sample.

Table 4 Internal reliability and retest reliability of 25 facets of the PID-5

Table 5 Hierarchical linear regression analysis of PID-5 trait facets to DSM-5 section II personality disorders

Supplementary table 1

Descriptive analysis for 25 facets and 5 domains of the PID-5 in undergraduate sample

	M±SD	Skewness	Kurtosis
**25factes**			
Anhedonia	0.76±.044	0.53	0.17
Anxiousness	1.21±0.51	0.23	-0.10
Attention Seeking	1.21±0.53	0.12	-0.05
Callousness	0.51±0.32	0.68	-0.01
Deceitfulness	0.73±0.39	0.58	0.02
Depressivity	0.56±0.38	0.10	1.10
Distractibility	1.02±0.48	0.10	-0.16
Eccentricity	0.70±0.48	0.64	0.13
Emotional Lability	0.99±0.54	0.28	-0.19
Grandiosity	0.86±0.42	0.27	-0.11
Hostility	0.91±0.40	0.20	-0.06
Impulsivity	0.87±0.49	0.47	0.04
Intimacy Avoidance	0.93±0.50	0.48	0.32
Irresponsibility	0.65±0.38	0.26	-0.46
Manipulativeness	0.82±0.44	0.31	-0.13
Perceptual Dysregulation	0.83±0.45	0.34	-0.20
Perseveration	1.06±0.44	-0.10	-0.02
restricted affect	1.04±0.40	0.24	0.11
Rigid Perfectionism	1.13±0.44	0.13	0.16
Risk Taking	1.26±0.38	0.04	0.27
Separation Insecurity	0.94±0.54	0.34	-0.28
Submissiveness	1.03±0.51	0.10	-0.27
Suspiciousness	0.83±0.33	0.17	0.40
Unusual Belief	0.71±0.48	0.55	-0.11
Withdrawal	0.79±0.45	0.40	-0.16
**5 domains**			
Negative Affect	1.06±0.42	0.13	-0.27
Detachment	0.81±0.37	0.30	-0.36
Antagonism	0.79±0.35	0.34	-0.30
Disinhibition	0.87±0.38	0.07	-0.40
Psychoticism	0.75±0.41	0.33	-0.43

Note: M=mean; SD= standard deviation.

Supplementary table 2

Descriptive analysis for 25 facets and 5 domains of the PID-5 in clinical patient sample

	M±SD	Skewness	Kurtosis
**25factes**			
Anhedonia	1.52±0.68	0.10	-0.52
Anxiousness	1.90±0.63	-0.33	-0.11
Attention Seeking	1.31±0.65	0.17	-0.41
Callousness	0.78±0.45	0.76	0.87
Deceitfulness	1.06±0.53	0.28	-0.34
Depressivity	1.34±0.69	0.22	-0.63
Distractibility	1.49±0.61	-0.13	-0.10
Eccentricity	1.28±0.67	0.30	-0.21
Emotional Lability	1.66±0.69	-0.10	-0.41
Grandiosity	1.00±0.56	0.42	0.14
Hostility	1.39±0.52	0.21	0.34
Impulsivity	1.24±0.62	0.39	-0.06
Intimacy Avoidance	1.19±0.64	0.52	-0.08
Irresponsibility	0.97±0.51	0.38	0.15
Manipulativeness	1.00±0.57	0.27	-0.10
Perceptual Dysregulation	1.20±0.61	0.21	-0.45
Perseveration	1.43±0.56	-0.02	0.29
restricted affect	1.27±0.46	0.07	-0.16
Rigid Perfectionism	1.47±0.54	0.14	-0.04
Risk Taking	1.20±0.02	0.15	0.03
Separation Insecurity	1.20±0.64	0.20	-0.40
Submissiveness	1.36±0.65	0.15	-0.03
Suspiciousness	1.19±0.48	0.44	0.53
Unusual Belief	1.01±0.65	0.57	0.08
Withdrawal	1.49±0.66	0.09	-0.43
**5 domains**			
Negative Affect	1.61±0.53	-0.17	-0.17
Detachment	1.43±0.56	0.15	-0.34
Antagonism	1.03±0.45	0.18	-0.08
Disinhibition	1.25±0.49	0.14	0.24
Psychoticism	1.19±0.57	0.33	0.11

Supplementary table 3

The t-test between undergraduate sample and personality disorders sample.

	M±SD		t
	**Undergraduate sample N=3550**	**Personality disorders sample N=125**	
**25 facets**	0.76±.44		
Anhedonia	1.21±.51	1.31±0.59	10.33**
Anxiousness	1.21±.53	1.99±0.50	17.00**
Attention Seeking	0.51±.32	1.46±0.58	5.34**
Callousness	0.73±.39	0.74±0.38	7.97**
Deceitfulness	0.56±.38	1.07±0.50	7.64**
Depressivity	1.02±.48	1.19±0.56	12.59**
Distractibility	0.70±.48	1.46±0.53	10.05**
Eccentricity	0.99±.54	1.41±0.61	12.76**
Emotional Lability	0.86±.42	1.62±0.57	12.83**
Grandiosity	0.91±.40	1.11±0.52	5.29**
Hostility	0.87±.49	1.37±0.44	12.51**
Impulsivity	0.93±.50	1.21±0.51	7.62**
Intimacy Avoidance	0.65±.38	1.24±0.60	5.70**
Irresponsibility	0.82±.44	0.90±0.44	7.02**
Manipulativeness	0.83±.45	1.06±0.53	5.10**
Perceptual Dysregulation	1.06±.44	1.48±0.52	15.41**
Perseveration	1.04±.40	1.60±0.48	13.17**
restricted affect	1.13±.44	1.36±0.42	8.81**
Rigid Perfectionism	1.26±.38	1.59±0.52	9.76**
Risk Taking	0.94±.54	1.16±0.45	-2.48**
Separation Insecurity	1.03±.51	1.23±0.61	5.77**
Submissiveness	0.83±.33	1.33±0.55	6.47**
Suspiciousness	0.71±.48	1.18±0.38	10.13**
Unusual Belief	0.79±.45	1.27±0.64	9.78**
Withdrawal	0.76±.44	1.37±0.57	11.29**
**5 domains**			
Negative Affect	1.61±0.53	1.65±0.43	15.20**
Detachment	1.43±0.56	1.32±0.47	11.72**
Antagonism	1.03±0.45	1.08±0.41	7.79**
Disinhibition	1.25±0.49	1.21±0.39	10.19**
Psychoticism	1.19±0.57	1.40±0.49	14.82**

Note: M=mean; SD= standard deviation

** P<0.001

Supplementary table4

Internal reliability and retest reliability of 25 facets of the PID-5

	Internal Reliability (undergraduate sample)		Internal Reliability (clinical patient sample)		Retest reliability
	α	MIC	α	MIC	
Anhedonia	0.78	0.31	0.88	0.48	0.53**
Anxiousness	0.82	0.33	0.87	0.43	0.77**
Attention Seeking	0.83	0.38	0.87	0.45	0.68**
Callousness	0.83	0.26	0.83	0.27	0.67**
Deceitfulness	0.78	0.25	0.79	0.28	0.70**
Depressivity	0.87	0.33	0.93	0.47	0.74**
Distractibility	0.83	0.35	0.87	0.43	0.76**
Eccentricity	0.91	0.43	0.93	0.50	0.81**
Emotional Lability	0.83	0.41	0.87	0.49	0.79**
Grandiosity	0.69	0.27	0.76	0.35	0.74**
Hostility	0.74	0.23	0.79	0.27	0.67**
Impulsivity	0.77	0.36	0.83	0.44	0.68**
Intimacy Avoidance	0.70	0.28	0.76	0.34	0.57**
Irresponsibility	0.68	0.23	0.71	0.26	0.64**
Manipulativeness	0.66	0.28	0.73	0.36	0.52**
Cognitive and Perceptual Dysregulation	0.81	0.26	0.87	0.35	0.79**
Perseveration	0.78	0.28	0.81	0.32	0.71**
restricted affect	0.55	0.15	0.53	0.14	0.59**
Rigid Perfectionism	0.77	0.26	0.80	0.28	0.70**
Risk Taking	0.76	0.19	0.82	0.24	0.57**
Separation Insecurity	0.74	0.29	0.78	0.34	0.61**
Submissiveness	0.67	0.34	0.77	0.45	0.61**
Suspiciousness	0.54	0.14	0.68	0.23	0.53**
Unusual Belief	0.77	0.30	0.83	0.37	0.79**
Withdrawal	0.84	0.35	0.89	0.45	0.78**

Note: α values are Cronbach's α coefficients, MICs describe mean inter-item correlations

**p < 0.01.

Supplementary table 6

Hierarchical linear regression analysis of PID-5 trait facets to DSM-5 section II personality disorders

DSM-IV PD to be predicted	Step	Variable entered	R^2	△ R^2	P	β	P
PPD	1		.30		.00		
		Hostility				.31	.00
		suspiciousness				.25	.00
		Intimacy avoidance				-.07	.00
		Unusual thoughts and beliefs				.14	.00
	2		.35	.05	.00		
		Anhedonia				.05	.02
		Anxiousness				.19	.00
		Attention seeking				.09	.00
		Depressivity				-.05	.04
		Impulsivity				-.06	.02
		Irresponsibility				-.07	.00
		Perceptual dysregulation				.07	.00
		Manipulativeness				.04	.03
SPD	1		.26		.00		
		restricted affect				.06	.00
		Withdrawal				.26	.00
		Intimacy avoidance				.22	.00
		anhedonia				.11	.00
	2		.29	.03	.00		
		Distractibility				-.08	.00
		Distractibility				-.08	.00
		Eccentricity				.07	.00
		Emotional lability				.05	.02
		Irresponsibility				-.09	.00
		Perceptual dysregulation				.10	.00
		Perseveration				.05	.03
		Separation insecurity				-.08	.00
		submissiveness				-.07	
		Unusual thoughts and belief				-.06	.01
STPD	1		.28		.00		
		Eccentricity				.14	.00
		Cognitive and perceptual dysregualtion				.06	.00
		Unusual thoughts and beliefs				.28	.00
		restricted affect				.04	.05
		withdrawal				.08	.00
		suspiciousness				.10	.00
	2		.33	.05	.00		
		Anxiousness				.10	.00
		Callousness				-.11	.00
		Distractibility				-.04	.04
		Hostility				.12	.00
		Impulsivity				-.07	.00
		Intimacy avoidance				-.04	.02
		Irresponsibility				-.10	.00
		Rigid perfectionism				.04	.02
BPD	1		.44		.00		
		Emotional lability				.25	.00
		Anxiousness				.17	.00
		Separation insecurity				.05	.00
		Depressivity				.17	.00
		Impulsivity				.09	.00
		Risk taking				.01	.42
		Hostility				.11	.00
BPD	2		.46	.02	.00		
		Anhedonia				.06	.00
		Attention seeking				.04	.01
		Callousness				-.06	.00
		Grandiosity				-.04	.04
		Intimacy avoidance				-.04	.01
		Cognitive and perceptual dysregulation				.18	.00
		Rigid perfectionism				-.04	.04
ASPD	1		.27		.00		
		Manipulativeness				.03	.14
		Deceitfulness				.17	.00
		Callousness				.04	.10
		Hostility				.06	.00
		Irresponsibility				.02	.28
		Impulsivity				.14	.00
		Risk taking				.32	.00
	2		.29	.02	.00		
		anxiousness				.05	.03
		Attention seeking				.05	.02
		Eccentricity				.09	.00
		Intimacy Avoidance				-.08	.00
		Rigid perfectionism				-.07	.00
		Separation Insecurity				-.04	.04
		submissiveness				-.08	.00
NPD	1		.24		.00		
		Grandiosity				.27	.00
		Attention Seeking				.29	.00
	2		.36	.12	.00		
		Anhedonia				.05	.00
		Anxiousness				.14	.00
		Callousness				-.08	.00
		Depressivity				-.05	.04
		Eccentricity				.04	.03
		Emotional lability				.06	.01
		Hostility				.17	.00
		Cognitive and perceptual dysregulation				.09	.00
		submissiveness				-.05	.00
		Suspiciousness				.08	.00
HPD	1		.31		.00		
		Emotional lability				.24	.00
		manipulativeness				.08	.00
		Attention seeking				.38	.00
	2		.35	.04	.00		
		Anhedonia				-.06	.01
		Anxiousness				.08	.00
		Distractibility				.08	.00
		Grandiosity				.07	.00
		Hostility				.05	.02
		Impulsivity				.07	.00
		restricted affect				-.08	.00
		Submissiveness				.04	.02
		Withdrawal				-.12	.00
APD	1		.33		.00		
		Anxiousness				.36	.00
		Anhedonia				.17	.00
		Withdrawal				.20	.00
		Intimacy avoidance				-.05	.00
	2		.38	.05	.00		
		Attention seeking				.08	.00
		Callousness				-.13	.00
		Depressivity				.07	.03
		Distractibility				.04	.04
		Hostility				.13	.00
		Restricted affect				.04	.04
		Risk taking				-.06	.00

DSM-IV PD to be predicted	Step	Variable entered	R^2	△ R^2	P	β	P
APD	2						
		Manipulativeness				-.08	.00
		Separation insecurity				.08	.00
		submissiveness				.08	.00
		suspiciousness				.05	.00
		Unusual belief and thoughts				-.08	.00
OCPD	1		.29		.00		
		Rigid perfectionism				.36	.00
		perseveration				.22	.00
		Intimacy avoidance				.06	.00
		restricted affect				.04	.00
	2		.33	.04	.00		
		Anxiousness				.10	.00
		Attention seeking				.08	.00
		Callousness				-.08	.00
		Deceitfulness				-.07	.00
		Eccentricity				.08	.00
		Hostility				.07	.00
		Irresponsibility				-.07	.00
		Cognitive and perceptual dysregulation				.09	.00
		suspiciousness				.05	.01
		Unusual belief and thoughts				-.07	.00
		withdrawal				.10	.00
DPD	1		.32		.00		
		submissiveness				.22	.00
		Separation insecurity				.25	.00
		anxiousness				.26	.00
	2		.37	.05	.00		
		Attention seeking				.06	.00
		Callousness				-.09	.00
		Distractibility				.08	.00
		Eccentricity				-.06	.00
		Emotional lability				.05	.02
		Hostility				.04	.05
		Irresponsibility				.09	.00
		Cognitive and perceptual dysregulation				.09	.00
		Perseveration				.06	.00
		Risk taking				-.09	.00
		Unusual belief and thoughts				-.07	.00

Note: Step 1 entered contributed facets and step 2 entered other correlated facets in regression analysis. The non-specified traits did not show in this table when failed statistic criteria($p<0.05$).
